# Epigenome-Wide Assessment of DNA Methylation in the Placenta and Arsenic Exposure in the New Hampshire Birth Cohort Study (USA)

**DOI:** 10.1289/ehp.1510437

**Published:** 2016-01-15

**Authors:** Benjamin B. Green, Margaret R. Karagas, Tracy Punshon, Brian P. Jackson, David J. Robbins, E. Andres Houseman, Carmen J. Marsit

**Affiliations:** 1 Department of Epidemiology, Geisel School of Medicine at Dartmouth, Lebanon, New Hampshire, USA; 2 Department of Pharmacology and Toxicology, Geisel School of Medicine at Dartmouth, Hanover, New Hampshire, USA; 3 Children’s Environmental Health and Disease Prevention Research Center at Dartmouth, Geisel School of Medicine at Dartmouth, Lebanon, New Hampshire, USA; 4 Department of Biological Sciences, and; 5 Department of Earth Sciences, Dartmouth College, Hanover, New Hampshire, USA; 6 Molecular Oncology Program, The DeWitt Daughtry Family Department of Surgery, University of Miami Miller School of Medicine, Miami, Florida, USA; 7 School of Biological and Population Health Sciences, College of Public Health and Human Sciences, Oregon State University, Corvallis, Oregon, USA

## Abstract

**Background:**

Arsenic is one of the most commonly encountered environmental toxicants, and research from model systems has suggested that one mode of its toxic activity may be through alterations in DNA methylation. In utero exposure to arsenic can affect fetal, newborn, and infant health, resulting in a range of phenotypic outcomes.

**Objectives:**

This study examined variation in placental DNA methylation and its relationship to arsenic exposure in 343 individuals enrolled in the New Hampshire Birth Cohort Study.

**Methods:**

Linear regression models using a reference-free correction to account for cellular composition were employed to determine CpG loci affected by arsenic levels.

**Results:**

Total arsenic measured in maternal urine during the second trimester was not associated with methylation in the placenta, whereas arsenic levels quantified through maternal toenail collected at birth were associated with methylation at a single CpG locus (p = 4.1 × 10–8). Placenta arsenic levels were associated with 163 differentially methylated loci (false discovery rate < 0.05), with 11 probes within the LYRM2 gene reaching genome-wide significance (p < 10–8). Measurement of LYRM2 mRNA levels indicated that methylation was weakly to moderately correlated with expression (r = 0.15, p < 0.06). In addition, we identified pathways suggesting changes in placental cell subpopulation proportions associated with arsenic exposure.

**Conclusions:**

These data demonstrate the potential for arsenic, even at levels commonly experienced in a U.S. population, to have effects on the DNA methylation status of specific genes in the placenta and thus supports a potentially novel mechanism for arsenic to affect long-term children’s health.

**Citation:**

Green BB, Karagas MR, Punshon T, Jackson BP, Robbins DJ, Houseman EA, Marsit CJ. 2016. Epigenome-wide assessment of DNA methylation in the placenta and arsenic exposure in the New Hampshire Birth Cohort Study (USA). Environ Health Perspect 124:1253–1260; http://dx.doi.org/10.1289/ehp.1510437

## Introduction

One hundred million individuals worldwide, 13 million of whom reside within the United States, are exposed to arsenic in drinking water at levels above the World Health Organization and U.S. Environmental Protection Agency recommended limit of 10 μg/L (U.S. EPA 2009). Arsenic exposure often originates from the use of unregulated wells for drinking water as well as from dietary sources, with levels of exposure varying widely depending on the population and region studied (EFSA 2009; Gilbert-Diamond et al. 2011; WHO 2011). Arsenic exposure during pregnancy is of special concern to fetal health because arsenic easily crosses the placenta, exposing the infant to levels comparable to those of the mother (Concha et al. 1998). Fetal exposure to arsenic has previously been linked to negative birth outcomes including a general increase in infant mortality (Rahman et al. 2007, 2010), low birth weight (Gelmann et al. 2013; Laine et al. 2015), poor infant neurodevelopment (Hamadani et al. 2010, 2011), as well as increased infant infection rates and alterations in their immune profile (Farzan et al. 2013; Nadeau et al. 2014).

Although several studies have investigated the associations between arsenic exposure and epigenetic alterations in both adult blood (Argos et al. 2015; Bailey et al. 2013; Liu et al. 2014; Seow et al. 2014) and cord blood (Kile et al. 2014; Koestler et al. 2013), there is a gap in our understanding of the role of maternal arsenic exposure as it relates to fetal DNA methylation on a genome-wide scale within the placenta. In existing genome-wide–scale studies, DNA examined from umbilical cord blood samples indicated differential methylation in specific regions across the genome, with variation enriched at loci within CpG islands (Koestler et al. 2013). In addition, employing a statistical model to estimate leukocyte proportions from DNA methylation array data (Houseman et al. 2012), an increase in the proportion of CD8^+^ T cells was described as a function of arsenic exposure (Koestler et al. 2013), a finding that was later replicated in a Bangladeshi population exposed to a wider range of arsenic (Kile et al. 2014).

The placenta may be an ideal candidate for investigations of fetal programming and environmental impacts on long-term health, because it mediates nutrient and waste exchange, regulates interaction with the maternal immune system, acts as a neuroendocrine organ, and regulates fetal exposure to exogenous compounds present in the maternal circulation, making it a master regulator for the fetal environment (Maccani and Marsit 2009; Novakovic and Saffery 2012). Recent studies have linked variation in DNA methylation in the placenta with a number of environmental exposures as well as newborn health outcomes including growth and neurobehavioral functioning (Appleton et al. 2015; Lesseur et al. 2014; Maccani et al. 2015; Simpkin et al. 2015). We hypothesized that maternal arsenic exposure can affect the function of the placenta through variation in the placental DNA methylation profile. To assess the relationship between arsenic and the placental epigenome, we examined samples originating from the New Hampshire Birth Cohort Study (NHBCS), where individuals have varying levels of arsenic exposure from use of unregulated private wells for drinking water as well from dietary exposure (Farzan et al. 2013). Genome-wide DNA methylation was measured in placenta samples, and the relationship between methylation and various biomarkers of maternal and fetal arsenic exposure, including maternal urine and toenail samples as well as placenta levels, was assessed. Further, we investigated how arsenic-associated variable DNA methylation was correlated with gene expression at identified regions. To our knowledge, this study presents the first genome-wide analysis of placental DNA methylation as it relates to arsenic exposure *in utero*.

## Materials and Methods

### Study Population

The study population consisted of 343 mother–infant pairs who were participants in the New Hampshire Birth Cohort Study, an ongoing prospective study initiated in 2009. Participants chosen for these analyses included all mothers recruited into the cohort from February 2012 through September 2013. All subjects provided written informed consent in accordance with the requirements of the Institutional Review Board of Dartmouth College. Briefly, mothers enrolled in the study were between 18 and 45 years of age, reporting use of a private, unregulated well at their home, pregnant with a singleton infant, and not planning to move.

### Sample Collection

Maternal urine samples were collected at approximately 24–28 weeks gestation following protocols described elsewhere (Gilbert-Diamond et al. 2011). Maternal toenail samples were obtained following delivery, as previously described (Davis et al. 2014). Following delivery, the placenta was biopsied adjacent to the cord insertion (to minimize heterogeneity), removing any maternal decidua, placed immediately in RNAlater (Life Technologies, Carlsbad, CA) and frozen at –80°C within 24 hr. DNA and RNA were extracted by using the RNA/DNA extraction kit (Norgen Biotek, Thorold, ON) subsequently quantified using the Qubit Fluorometer (Life Technologies) and stored at –80°C.

### Arsenic Quantification

Arsenic levels were quantified within maternal urine (collected between 24 and 28 weeks gestation), maternal postpartum toenail samples and placenta from 342 individuals with downstream analysis conducted on samples with values above the detectable limit. Arsenic in all three tissues was quantified using inductively coupled plasma dynamic reaction cell–mass spectrometry. The protocol has been described previously (Davis et al. 2014; Punshon et al. 2015). A total of 285 samples had detectable placental arsenic data, and for these the detection limit for arsenic in placenta was 0.0148 ng/g. Total urinary arsenic was calculated as the sum of arsenate, arsenite, monomethylarsonic acid (MMA), and dimethylarsinic acid (DMA). Detection limits for the four individual arsenic species ranged from 0.10 to 0.15 mg/L and 144, 138, 59, and 3 of 271 samples with available data were below detection limit for arsenate, arsenite, MMA, and DMA, respectively. For samples with one of these individual species below the detection limit, we assigned values equal to the detection limit divided by the square root of two (Helsel 1990). Arsenic was detected in 257 of the maternal toenail samples. We further performed Pearson correlation tests on log_10_-transformed arsenic values from the three tissues.

### DNA Methylation Quantification and Normalization

Isolated DNA underwent bisulfite modification using the EZ Methylation kit (Zymo Research, Irvine, CA). Samples of bisulfite-modified DNA were then randomized across several plates and assessed for epigenome-wide DNA methylation levels using the Infinium HumanMethylation450 (450K) Bead Chip (Illumina, San Diego, CA) profiling methylation status for approximately 486,000 CpG loci. The microarrays were processed at the Biomedical Genomics Center at the University of Minnesota (Minneapolis, MN). Data was assembled using the BeadStudio methylation software package (Illumina) and then processed using the minfi package in R (R Core Team 2014). For our analysis, we removed all probes that were present on either the X or Y chromosome, have previously been identified as cross-hybridizing with other genomic locations (Chen et al. 2013), or contained a single nucleotide polymorphism. In addition, probes identified as having detection *p-*values > 0.01 in at least one sample were removed. This resulted in 344,348 autosomal probes from 343 unique samples being included within our analysis.

Data were normalized using the functional normalization (funNorm) protocol within the minfi package as specified by the software authors (http://bioconductor.org/packages/release/bioc/html/minfi.html). A principal-component analysis (PCA) was then used to determine whether technical aspects of our design (bisulfite conversion plate or microarray slide) might be influencing underlying variation within the data set. We examined the two principal components accounting for the largest degree of variation and found that plate was associated with a significant portion of the variation. To correct this, we adjusted our data for this effect using the “ComBat” method (Johnson et al. 2007), and again used PCA to assure this bias was corrected. Raw and processed data have been provided via the NCBI Gene Expression Omnibus (GEO) accession number GSE71678.

### Cell Mixture Deconvolution and Analysis

Using normalized and ComBat adjusted methylation data, we proceeded to investigate the relationship between placental methylation and measured levels of arsenic exposure. Of the 343 samples within our study, 285, 257, and 271 had available placenta, toenail, and total urinary arsenic measurements, respectively. These subsets were used to determine the relationship between arsenic and methylation. The relationships between methylation and each measure of arsenic were evaluated via epigenome-wide association studies (EWAS) using the R package RefFreeEWAS, which fits a series of linear models and subsequently accounts for cell proportion variability within tissue samples without a reference set (Houseman et al. 2014). Although the reference-free approach does not require *a priori* knowledge of cell composition within a tissue to accomplish the adjustment, placenta tissue is likely a combination of variously differentiated trophoblasts, stromal cells including fibroblasts, and mesenchymal cells including Hofbauer and immune cells (Wang and Zhao 2010). Using DNA methylation as the dependent variable, we performed an EWAS with arsenic levels in placental, maternal urine, or maternal toenail as the explanatory variable, while also including maternal age, gestational age, and infant sex as potential confounding covariates within the model. Maternal and gestational ages were modeled as continuous variables. A separate EWAS was performed for each arsenic measurement from the three tissues. Genome-wide significance based on a Bonferroni threshold was considered at *p* < 1.0 × 10^–7^, though we report all loci that passed an FDR (false discovery rate) correction of *q* < 0.05. All *p*-values reported are the product of the multiple comparisons correction.

The RefFreeEWAS method produces an effect estimate for each of the CpG loci investigated and returns results for both a covariate-only–adjusted (β) and a covariate- and cell-mixture–adjusted (β*) model. Those loci exhibiting the large differences between the β and β* estimates would represent those most confounded by differences in cell subpopulation proportions. By linking the genes associated with these highly confounded CpG sites to gene ontology (GO) terms, we can make an indirect assessment of the representation of these genes within specific functions or pathways, and then infer the types of cellular functions and thus types of cells, most likely demonstrating altered subpopulations by arsenic exposure. To decide which loci to include, we calculated the difference (δ) between β* and β for each locus, and used the resulting value and standard error to derive a *p*-value for δ, with a null hypothesis of β* = β. We considered all loci with *p* < 0.10 to have undergone significant cell mixture corrections while including placental arsenic levels within the model. A Cochran–Mantel–Haenszel test was then employed to determine associations between these loci and GO term pathways while accounting for the locus’s relation to a CpG island as well as position relative to a gene. This would then allow us to indirectly determine how exposure to arsenic *in utero* may be altering cellular composition within the placenta based upon pathways overrepresented among these loci.

### Validation of Previously Identified Loci Associated with Arsenic Exposure

Several recent papers have investigated the role of arsenic exposure on an epigenome-wide scale. Although none of these reports have examined placenta-specific DNA methylation, the use of an identical or similar platform does allow for some comparisons to be made in the findings. These studies investigated the effects of arsenic on DNA methylation as measured in both maternal (Argos et al. 2015; Liu et al. 2014; Seow et al. 2014) and cord (Kile et al. 2014; Koestler et al. 2013; Rojas et al. 2015) blood or, in a single study, urothelial carcinomas (Yang et al. 2014). We examined these loci by linear regression model using methylation as the explained variable and placental arsenic as the explanatory variable.

### LYRM2 Gene Expression and Analysis

The expression status of LYRM2 was examined, because the methylation status of 11 neighboring CpG loci within the promoter of this gene were found to be genome-wide significantly associated with arsenic exposure. Placental RNA samples from a subset of 96 individuals, selected from the highest and lowest tertile of average methylation for the gene *LYRM2,* were used for gene expression analysis. Due to sample failure, we were able to collect expression data from 93 individuals. For each sample, cDNA as well as a non-template control were produced using the iScript cDNA master mix (Bio-Rad, Hercules, CA) as specified within manufacturer’s instructions and stored at –4°C. mRNA levels were quantified using the iQ SYBER Green Supermix (Bio-Rad) on the CFX Connect Real-Time PCR Detection System (Bio-Rad) per manufacturer’s instructions. Primers specific to *LYRM2* were used from the TaqMan Gene Expression Assays kit (Life Technologies, Cat #4351372) following recommended PCR (polymerase chain reaction) conditions. Expression levels were normalized against the housekeeping gene succinate dehydrogenase (*SDHA*) using TaqMan Gene Expression Assay primers (Life Technologies). We selected *SDHA* for our housekeeping gene because it has previously been shown to be the most effective for use in placental gene expression studies due to stable expression across samples (Meller et al. 2005).

Pearson correlation coefficients were calculated to determine the relationship between DNA methylation within the *LYRM2* gene and expression levels. *LYRM2* methylation was input as the average β value for the 11 probes that were identified by our previous modeling. All statistical analyses were conducted within the R statistical package, version 3.1.0 (R Core Team 2014).

## Results

The characteristics of the 343 mother–infant pairs examined in this analysis are presented in Table 1. Arsenic levels in all media had a broad range across individuals with a general skew toward the right. Mothers were on average approximately 31.5 years of age at the time of birth, with a body mass index (BMI) of 25.6 calculated from prepregnancy weight. There was an approximately even distribution of male and female infants within the study population, with an average of 39.4 weeks gestation and 3.4 kg birth weight. We also determined the relationship between the measures of arsenic for the three tissues as calculated from the 176 individuals with values for all three biological samples. There was no correlation between the measures of arsenic within the placenta and maternal toenail samples (*r* = 0.03), or between placenta and urine (*r* = 0.03), and only a weak correlation between maternal toenail samples and urine (*r* = 0.19).

**Table 1:** Demographic and clinical characteristics of the study population.

Characteristic	Value
Maternal age (years)	31.5 ± 4.9
Prepregnancy maternal BMI	25.6 ± 5.4
Gestational age (weeks)	39.4 ± 1.6
Infant birth weight (kg)	3.4 ± 0.5
Male	183 (53.5)
Female	159 (46.5)
Placenta arsenic (μg/kg)^*a*^	0.82 (0.47–1.17)
Toenail arsenic (μg/kg)^*b*^	0.05 (0.026–0.073)
Urinary total arsenic (μg/L)^*c*^	3.76 (1.87–5.65)
Values are presented as mean ± SD, *n* (%), or median (interquartile range). ^***a***^285 of the 343 mother–infant pairs had available placenta arsenic data. ^***b***^257 of the 343 mother–infant pairs had available post-partum toenail arsenic data. ^***c***^271 of the 343 mother–infant pairs had available urinary arsenic data at 24–28 weeks gestation.	

**Figure 1:**
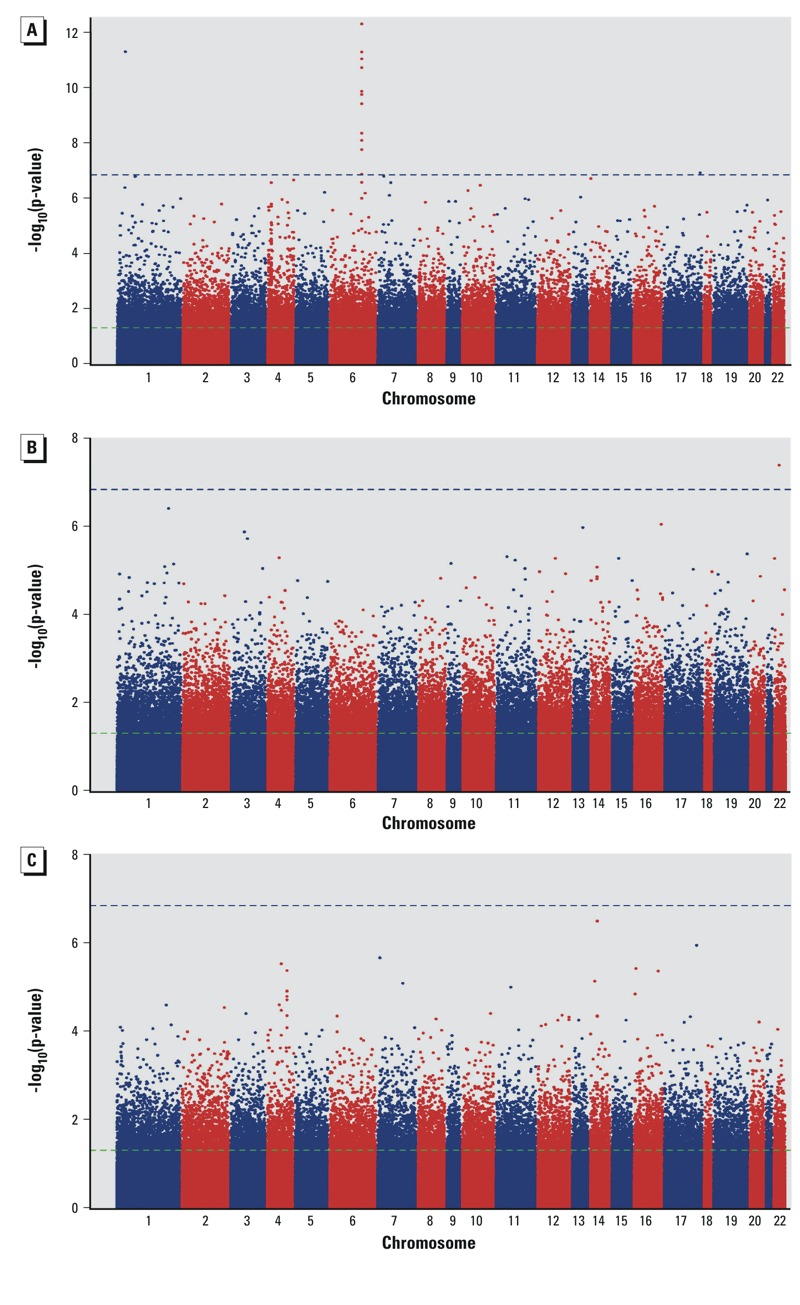
Manhattan plots of epigenome-wide association results for arsenic concentrations within the placenta (*A*), maternal toenail (*B*), and maternal urine (*C*). The horizontal green dashed line represents unadjusted significance threshold (*p* < 0.05), and the blue dashed line represents Bonferroni-adjusted significance threshold (*p* = 1.0 × 10^–7^).

**Figure 2:**
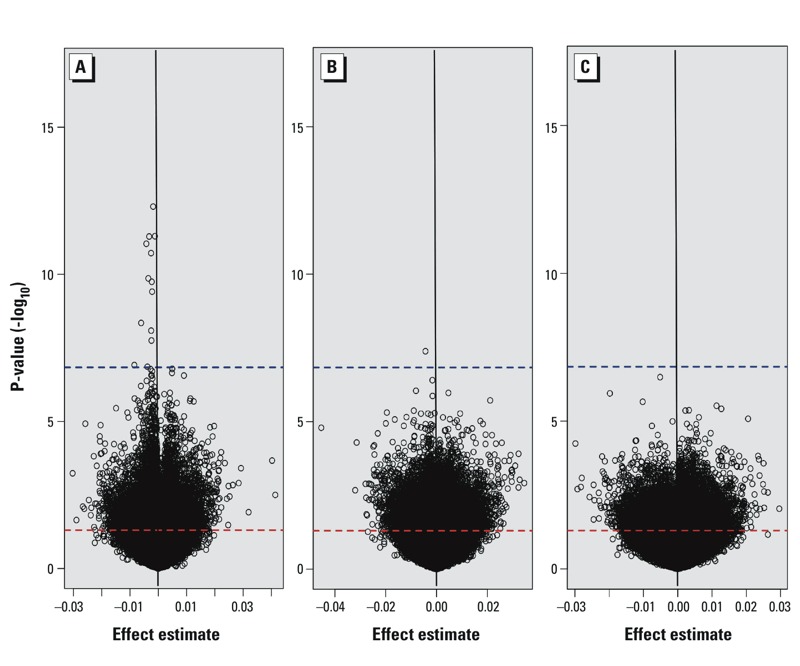
Volcano plots of the associations between placenta DNA methylation at 344,348 loci and placental (*A*), toenail (*B*), and total urinary (*C*) arsenic levels following adjustment for sample cellular composition. The threshold for *p* < 0.05 is indicated by a dashed red line, and the threshold for Bonferroni significance (*p* = 1.0 × 10^–7^) is indicated by a dashed blue line.

Focusing on our study, 163 CpG dinucleotides demonstrated significant associations (*q* < 0.05) with placental arsenic concentration (see Excel File Table S1). Of those, 13 attained genome-wide significance following multiple comparisons correction with the more stringent Bonferroni correction method (Figure 1A). Among these probes, 1 (*cg08640609*) tracked to calmodulin-binding transcription activator-1 (*CAMTA1*), 1 (*cg01777586*) to coiled-coil domain containing-57 (*CCDC57*), and 11 to LYR-motif containing 2 (*LYRM2)* (Table 2). Only 1 site (*cg01021483*), within the 3´UTR of mini-chromosome maintenance complex component 5 (*MCM5*), was genome-wide significantly (*q* < 0.05, *p* = 1.76 × 10^–8^) associated with toenail arsenic (Figure 1B). No loci demonstrated significant associations to total urinary arsenic (Figure 1C). Estimate effects and corresponding *p*-values were plotted for all three measures of arsenic exposure (Figure 2), and the majority of loci with methylation demonstrating significant associations with placental arsenic were hypomethylated with increasing arsenic exposure. None of the loci demonstrating a relationship between placental arsenic and DNA methylation showed any association when urinary or toenail arsenic were used as the explanatory variable after correcting for multiple comparisons (see Excel File Table S2).

**Table 2:** CpG loci identified as genome-wide significantly associated (Bonferroni *p* ≤ 0.05; *p* < 1 x 10^–7^) with placental arsenic levels following cell composition adjustment.

Loci	Coefficient^*a*^	FDR *q*-value	Bon *p*-value	Chromosome	Position	Gene
cg01953134	–0.0018	1.73E-07	1.73E-07	6	90348409	LYRM2
cg08640609	–0.0012	5.98E-07	1.75E-06	1	7764642	CAMTA1
cg27642643	–0.0032	5.98E-07	1.79E-06	6	90348450	LYRM2
cg11240327	–0.0042	7.92E-07	3.17E-06	6	90348503	LYRM2
cg10392378	–0.0026	1.32E-06	6.59E-06	6	90348606	LYRM2
cg21621759	–0.0035	7.94E-06	4.76E-05	6	90348302	LYRM2
cg03200341	–0.0023	8.87E-06	6.21E-05	6	90348309	LYRM2
cg21264329	–0.0022	1.68E-05	1.34E-04	6	90348123	LYRM2
cg04843946	–0.0061	1.73E-04	1.56E-03	6	90348009	LYRM2
cg02725437	–0.0025	2.84E-04	2.84E-03	6	90348314	LYRM2
cg25719236	–0.0024	5.60E-04	6.16E-03	6	90348382	LYRM2
cg01777586	–0.0085	3.49E-03	4.19E-02	17	80163174	CCDC57
cg25430506	–0.0038	3.68E-03	4.79E-02	6	90348385	LYRM2
Abbreviations: Bon, Bonferroni; FDR, false discovery rate. A complete list of loci may be found in Excel File Table S2. ^***a***^The difference in DNA methylation β value (0–1 scale) for every 1 unit (μg/kg) increase in placental arsenic.						

**Figure 3:**
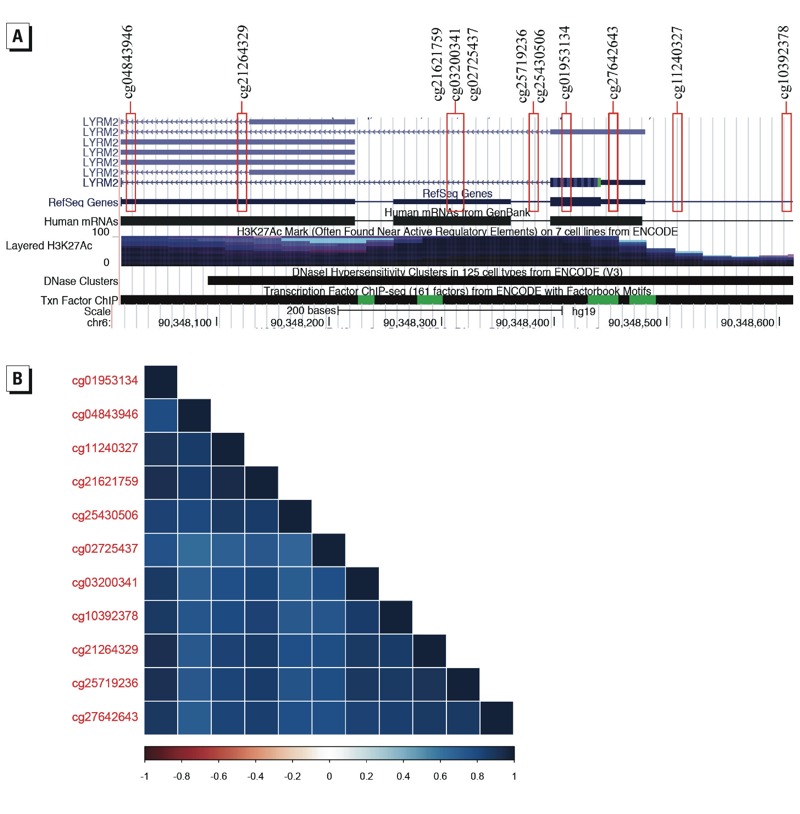
(*A*) Genomic tracks from the UCSC Genome Browser (University of California Santa Cruz 2015) for the region containing the 11 *LYRM2* loci identified as significantly associated with placental arsenic levels. (*B*) Correlelogram of Pearson correlation test of methylation at these 11 CpG loci, with corresponding heatmap indicating the value of *r* between –1 and 1.

**Figure 4:**
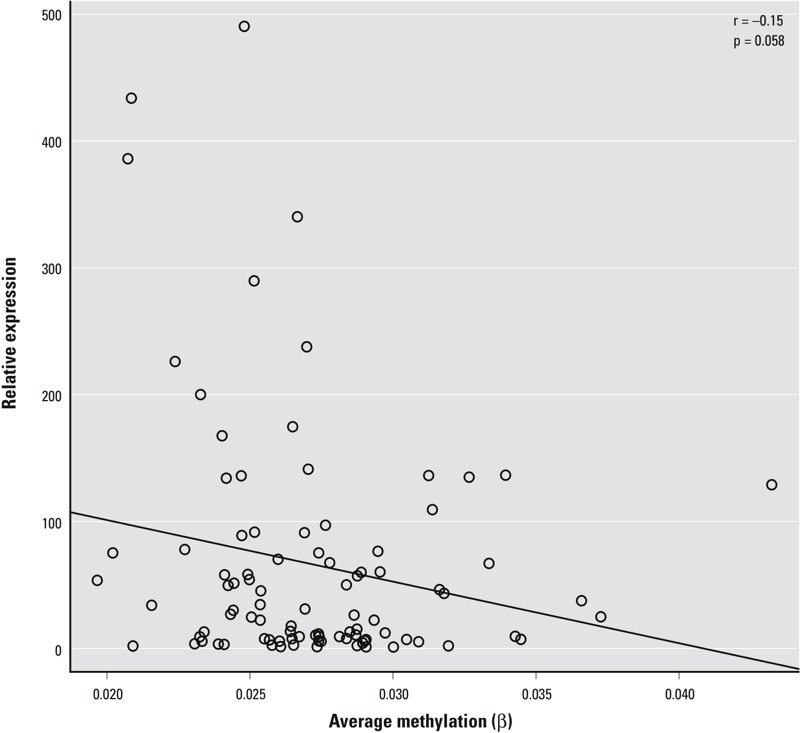
Scatter plot of the average methylation across the 11 significant *LYRM2* sites and relative expression of *LYRM2* mRNA for 93 individuals with the associated Pearson correlation. Individuals were selected from those within the highest and lowest tertiles of average *LYRM2* methylation.

The 11 probes associated with *LYRM2* were in the promoter region within approximately 600 bp of one another. These probes all showed a negative relationship with arsenic, demonstrating a decreased level of methylation as arsenic exposure increased. The methylation β-values displayed a strong positive correlation between each of the eleven sites, allowing downstream analysis to focus on mean methylation levels for these sites in order to reduce the number of comparisons being made (Figure 3). In a subsample of 93 individuals from the highest and lowest tertile of methylation for *LYRM2* loci, we observed a moderate negative correlation (*r* = –0.15) between *LYRM2* expression within the placenta and mean promoter methylation that approached (*p* = 0.058) statistical significance (Figure 4).

Due to the use of the RefFreeEWAS methodology, in addition to examining CpG loci with variable methylation most associated with placental arsenic exposure independent of cellular mixture, we can also identify those loci whose effect estimates are changed the most when cell mixture is controlled. Of the 344,348 loci analyzed from the methylation array, 10,596 sites showed substantial (Bonferroni corrected, *p* < 0.10) correction based upon the δ estimate, representing the difference in effect estimates of placental arsenic on methylation with and without cell mixture control. A total of 319 GO terms demonstrated significant overrepresentation (Bonferroni corrected, *p* < 0.05) among loci that were most affected by cellular subpopulation correction. The top 20 GO terms overrepresented amongst the genes associated with these loci are provided in Table 3.

**Table 3:** GO-term pathways (Gene Ontology Consortium 2015) significantly associated (Bonferroni < 0.05) with CpG loci with an altered effect estimate due to cellular proportion estimates.

GO ID	Adjusted *p*-value*	Test estimate**	GO pathway
9952	2.93E-32	3.267583	Anterior/posterior pattern specification
45944	1.23E-24	1.620397	Positive regulation of transcription from RNA polymerase II promoter
1764	8.85E-20	2.619747	Neuron migration
8045	3.16E-18	4.867233	Motor neuron axon guidance
48704	3.32E-17	3.356625	Embryonic skeletal system morphogenesis
45666	9.08E-17	2.780646	Positive regulation of neuron differentiation
7268	5.81E-16	1.74411	Synaptic transmission
122	6.65E-16	1.555492	Negative regulation of transcription from RNA polymerase II promoter
21884	9.60E-16	7.876803	Forebrain neuron development
7389	2.01E-15	3.83696	Pattern specification process
31018	7.34E-15	3.799679	Endocrine pancreas development
35115	2.13E-14	3.351906	Embryonic forelimb morphogenesis
44267	6.43E-14	0.3915227	Cellular protein metabolic process
21983	7.84E-14	3.640762	Pituitary gland development
1657	9.08E-14	3.162291	Ureteric bud development
21913	1.29E-13	7.137178	Regulation of transcription involved in ventral spinal cord interneuron specification
21912	1.36E-13	8.044255	Regulation of transcription involved in spinal cord motor neuron fate specification
9725	1.36E-13	3.760404	Response to hormone
6351	1.78E-13	1.351092	Transcription, DNA-templated
21797	2.49E-12	5.640727	Forebrain anterior/posterior pattern specification
*Adjusted Bonferroni *p*-value to account for multiple comparisons. **χ^2^_MH_.			

In the present study, we evaluated previously reported loci from other arsenic based epigenome-wide studies (Argos et al. 2015; Kile et al. 2014; Liu et al. 2014; Rojas et al. 2015; Seow et al. 2014; Yang et al. 2014) using a lookup approach to determine whether they presented a similar sensitivity in the placenta. In total, 3,393 CpG loci highlighted from these publications were also investigated within our array, though the majority of these loci were identified within a single study [3,316 from Rojas et al. (2015)]. Associations were identified for 293 of these CpG sites (*p* < 0.05, no adjustment for multiple comparisons) within our data set. Again, the majority of these were from the single Rojas et al. (2015) study, though 6 of the loci identified were from the other studies examined (see Excel File Table S3).

## Discussion

In this study we examined the association between DNA methylation within the placenta and arsenic exposure measured via three surrogate tissues. Arsenic measurements from maternal urine (median, 3.76 μg/L) were comparable with prior subsets examined from within this cohort (median, 4.1 μg/L) (Koestler et al. 2013), though they are lower than values obtained from a highly exposed Bangladeshi population (median, 12 μg/L) (Kile et al. 2014) as well as a representative sampling of the National Health and Nutrition Examination Survey participants (median, 4.2 μg/L) (Kuo et al. 2015). In addition, arsenic levels from the toenail, a measurement of long-term exposure was comparable, though slightly lower [interquartile range (IQR) = 0.026 μg/g to 0.073 μg/g] than values from the Coronary Artery Risk Development in Young Adults Study (IQR = < 0.0649 μg/g to > 0.1442 μg/g). Although there are no studies with comparable measurements of arsenic within the placenta, the arsenic values for urine and toenail samples from this cohort are within the range of other reported values, suggesting that the values we measured within the placenta would also be generalizable to exposure within other U.S.-based cohorts. A prior study in this cohort examining a much larger sample (*n* = 766) observed a significant relationship between placental arsenic levels and maternal urine or toenail (*r*_s_ = 0.11 and 0.12, respectively) (Punshon et al. 2015). In the present study, we saw only weak if any correlations between the biomarkers, likely due to the reduced sample size examined. Urinary arsenic may be considered a measurement of acute, recent exposure to arsenic (within the 24–28 weeks gestation during which samples were taken), in contrast with toenail samples, which give a view of more long-term exposures and storage within this tissue. The placenta likely represents a measurement of arsenic exposure and deposition within the window of the pregnancy. Thus, our disparate findings of variable DNA methylation by each of these biomarkers are not unexpected.

Using placental tissue from 343 mother–infant pairs, we identified that variation in DNA methylation of 163 CpG loci was statistically associated with arsenic levels at a 5% FDR. Among these 163 sites, 15 within the promoter region of *LYRM2* were linked to placental arsenic exposure burden, with 11 of these sites maintaining significance after the more conservative Bonferroni multiple tests correction. Further, we provide evidence that the loci identified within the *LYRM2* promoter region may influence gene transcription, consistent with the canonical role of promoter DNA methylation on gene transcription potential. Samples with the highest levels of methylation showed a trend toward muted expression levels, whereas samples with low methylation displayed a range of expression, though contained a high proportion of individuals with the highest levels of expression. The variability in the relationship between methylation and expression observed in this data, particularly at the lowest extents of DNA methylation, is expected, because the absence of methylation does not guarantee high expression levels, but instead endows a gene with the potential for expression. In addition, although DNA methylation is a major contributor to regulation of gene expression, there is not always a guaranteed relationship between these two biological measurements. Because of this, it is important to examine the relationship between identified differentially methylated regions and the expression of the associated gene, and to highlight the strength of the nearly significant relationship identified within this study.

*LYRM2* is a member of the LYR-motif–containing family of genes. Although LYRM2 currently lacks a known function within the placenta or other tissues, several members of this gene family have been ascribed an important role in iron–sulfur cluster (ISC) protein production (Angerer 2015; Maio et al. 2014). Specifically, researchers using a high-throughput yeast two-hybrid approach described an interaction between the C-terminus of the ISC chaperone protein, HSC20, and proteins bearing the LYR motif (Maio et al. 2014), suggesting that this motif targets proteins for ISC delivery. This result built upon previous reports linking LYRM4 (also known as ISD11) as a binding partner to NFS1 cysteine desulfurase (NFS1), a sulfur donor in ISC biogenesis (Shi et al. 2009). Deficits in ISC assembly manifest in several different forms in human health and disease including myopathy and neurodegenerative disease (Rouault 2012; Rouault and Tong 2008). In addition, several other members of the LYR superfamily are associated with the electron transport chain, specifically in the assembly of complex subunits I, II, III, and V (Angerer 2015). Although it is currently speculative to insert LYRM2 into these processes, the proportion of LYR-motif–containing genes associated with both the electron transport chain and ISC biogenesis would strongly suggest a role for LYRM2. Data from these prior publications would also suggest that proteins containing the LYR motif are targets of ISC delivery (Angerer 2015; Maio et al. 2014). This is intriguing because iron sulfur cluster containing proteins can act as a reductase involved in arsenic metabolism in anaerobic organisms (Saltikov and Newman 2003). Although no studies to our knowledge have implicated ISC proteins in eukaryotic arsenic metabolism, these findings may still potentially link arsenic to alterations in a gene with functional implications in the response to the exposure.

Although most loci identified resided within the *LYRM2* promoter, two CpG sites linked to separate genes were also found to be associated with placenta arsenic exposure. One site is located within the promoter region of *CAMTA1,* a member of the highly conserved family of transcription factors associated with calcium signaling through a direct binding to calmodulin (Bouché et al. 2002). Micro-rearrangements of this region have been implicated in congenital ataxia (Thevenon et al. 2012), whereas deletions have been associated with the progression of neuroblastomas (Katoh and Katoh 2003). The second site is located within the *CCDC57* promoter. Although the function of this gene is unclear, it has been identified to play a role in the interactome of nutritional metabolomics (Shin et al. 2014). In addition, overexpression of MCM5, identified here as associated with maternal toenail arsenic levels, has previously been identified in a variety of human cancers (Gakiopoulou et al. 2007; Guida et al. 2005; Liu et al. 2007). The biological implications of these genes are unclear as they relate to arsenic toxicity and arsenic-related birth outcomes, and further work is required to elucidate mechanistic involvement of these genes and to assess whether the methylation identified has any functional impact on these genes.

In addition to identifying regions affected by arsenic exposure, we also investigated the potential role of arsenic on alterations to placental cellular composition by examining which loci demonstrated effect estimates undergoing substantial change when cellular composition was included in the model. Such analyses of changes in blood cell composition based on estimates derived from DNA methylation data have been performed in studies of cancer as well as maternal arsenic and mercury exposures and infant umbilical cord blood (Cardenas et al. 2015; Kile et al. 2014; Koestler et al. 2013). We could not accomplish this directly because we could not directly examine cellular subpopulations based on histologic or morphologic criteria, and there is currently no reference set of DNA methylation data for placenta cell subsets, such as those that exist for blood samples (Reinius et al. 2012). Instead, we used a computational approach to examine whether the loci with the greatest change in effect estimate due to a correction for cell mixture were overrepresented among cellular pathways that could inform on specific cellular functions indicative of specific cell population, similar to analyses recently undertaken (Houseman et al. 2015). Our analysis noted (Table 3) that loci affected by arsenic-associated cellular composition changes were significantly associated with morphogenic and developmental processes such as anterior/posterior pattern specification, embryonic skeletal system morphogenesis, regulation of neuron differentiation, and forebrain neuron development, among others, suggesting potential important effects on placental development.

Previous investigations have also focused on the role of arsenic exposure in altering epigenetic signatures from a number of tissues, including both adult (Argos et al. 2015; Liu et al. 2014; Seow et al. 2014) and cord blood (Kile et al. 2014; Koestler et al. 2013; Rojas et al. 2015) and, in a single study, urothelial carcinomas (Yang et al. 2014). We compared the findings from those studies with our data and found an overlap in 293 of 3,393 previously identified differentially methylated loci. We did not perform a correction for multiple testing to the resulting *p*-value because this was a targeted lookup of association rather than a major exploratory analysis. Should these sites have been investigated without the *a priori* knowledge of the previous associations, the significance would not have held up following the epigenome-wide correction for multiple tests. Nonetheless, the associations between placental arsenic and the previously identified sites do lend a modest level of validation to their results. In addition, the significance seen in our study for these loci is of note in that none of these prior studies used placental tissue, suggesting there may be, to a certain degree, conservation across tissue types of the role of arsenic on methylation levels of a small proportion of genomic regions.

This study has several potential limitations. Our samples were collected from placentas at term, reducing the ability to infer how arsenic-associated methylation variability plays a role throughout development. Although this is one of the largest studies to date, linking maternal exposures to infant epigenetic features, we are still limited by our sample size in our ability to observe robust associations with potentially greater variability or small effect sizes. It will also be important, as additional populations begin to assess placenta DNA methylation, to replicate these findings in additional cohorts. With this in mind, however, it is important to note the many strengths of the current study. Our study offers perspective on a relatively healthy population exposed to arsenic levels in line with that of the general U.S. population. In addition, the measurement of arsenic levels within the placenta gives our study a direct assessment of likely arsenic levels present during gestation, rather than through the use of a proxy tissue.

## Conclusions

Arsenic exposure *in utero* has been implicated in a myriad of negative birth outcomes in infants, yet the mechanisms by which these changes occur are poorly understood. Here we found that arsenic exposure as measured within the placenta is associated with variation in placental DNA methylation, with a particularly strong association found with methylation of *LYRM2.* Data to replicate this study were unavailable. As we increase our understanding of the role of arsenic in infant health, should the results presented here be confirmed by other studies these data may highlight the role of the placenta in regulating arsenic exposure during pregnancy.

## Supplemental Material

(86 KB) ZIP
